# Disease Activity in Psoriatic Arthritis: Comparison of the Discriminative Capacity and Construct Validity of Six Composite Indices in a Real World

**DOI:** 10.1155/2014/528105

**Published:** 2014-05-20

**Authors:** Fausto Salaffi, Alessandro Ciapetti, Marina Carotti, Stefania Gasparini, Marwin Gutierrez

**Affiliations:** ^1^Clinica Reumatologica, c/o Ospedale C. Urbani-ASUR Marche Area Vasta 2, Università Politecnica delle Marche, Via dei Colli, 52 Jesi, 60035 Ancona, Italy; ^2^Dipartimento di Scienze Radiologiche, Università Politecnica delle Marche, Ancona, Italy

## Abstract

*Objective*. To compare, “in a real world,” the performance of the most common composite activity indices in a cohort of PsA patients. *Methods*. A total of 171 PsA patients were involved. The following variables were evaluated: peripheral joint assessment, patient reported of pain, physician and patient assessments of disease activity, patient general health status, dactylitis digit count, Leeds Enthesitis Index, Health Assessment Questionnaire (HAQ), physical and mental component summary score of the Medical Outcome Survey (SF-36), Psoriasis Area and Severity Index (PASI), Dermatology Life Quality Index, C-reactive protein (CRP), and erythrocyte sedimentation rate (ESR). To measure the disease activity, the Disease Activity Score (DAS28-ESR and DAS28-CRP), Simple Disease Activity Index (SDAI), Composite Psoriatic Disease Activity Index (CPDAI), disease activity in psoriatic arthritis (DAPSA), and Psoriatic Arthritis Disease Activity Score (PASDAS) have been calculated. The criteria for minimal disease activity (MDA) and remission were applied as external criterion. *Results*. The ROC were similar in all the composite measures. Only the CPDAI showed less discriminative ability. There was a high degree of correlation between all the indices (*P* < 0.0001). The highest correlations were between DAPSA and SDAI (rho = 0.996) and between DAPSA and DAS28-CRP (rho = 0.957). CPDAI, DAPSA, and PASDAS had the most stringent definitions of remission and MDA category. DAS28-ESR and DAS28-CRP had the highest proportions in remission and MDA. *Conclusions*. Although a good concurrent validity and discriminant capacity of six disease activity indices were observed, the proportions of patients classified in the disease activity levels differed. In particular, the rate of patients in remission was clearly different among the respective indices.

## 1. Introduction

Psoriatic arthritis (PsA) is a chronic inflammatory disease with widely variable intra- and interindividual clinical course and outcome. Its heterogeneity is such that the term “psoriatic disease” has been recently suggested to encompass the involvement of different tissue and organ levels. The prevalence among patients with psoriasis was reported as approximately 6.2% to 34.4% [1, 2]. In Italy, it has been estimated to be 36% in psoriatic subjects [3] and 0.42% in general population [4].

To date, it is largely known that an early and aggressive control of disease activity results in significantly better clinical, functional, and radiographic outcomes in patients with rheumatoid arthritis (RA) [5]. Although a similar paradigm of “treating-to-target” or “minimal disease activity” (MDA) has not yet been carefully established for PsA, it is clear that it is becoming the current challenge in the management of PsA [6, 7].

In RA, the ability to achieve a tight control is proportional to quantify the disease activity/severity by composite indices such as the Disease Activity Score (DAS) which assesses exclusively the joint involvement [8]. The heterogeneity of PsA that includes a possible combination of axial disease, peripheral arthritis, or specific features, such as enthesitis and dactylitis, as well as extra-articular manifestations, makes its global assessment that represents a significant challenge for clinical metrology difficult. Further, the combination of peripheral joint and axial and skin manifestations of PsA can have a tremendous impact on patient function, well-being, and health-related quality of life (HRQOL), even if the peripheral joint damage is more severe in RA [9, 10].

Although the peripheral involvement of PsA shares some clinical characteristics with RA, PsA shows some additional distinct features. Nevertheless, composite indices developed for RA, such as DAS 28-joint count and Simple Disease Activity Index (SDAI) [11], have been largely used to determine both disease activity and treatment response in PsA patients.

Over the last few years different tools to be used for measuring the disease activity in patients with PsA have been identified by the International Group for Research in Psoriasis and Psoriatic Arthritis (GRAPPA) and the Outcome Measures in Rheumatology Clinical Trials (OMERACT) [12]. Preliminary work has been undertaken in developing a more comprehensive disease activity instrument for psoriatic disease [12, 13]. The GRAPPA recently proposed the Composite Psoriatic Disease Activity Index (CPDAI) which classifies the PsA into mild, moderate, and severe taking into account the assessment of different domains such as peripheral arthritis, skin disease, spinal disease, enthesitis, and dactylitis [14]. The CPDAI assigns a score of 0–3 to each of the 5 domains of PsA based on disease activity and impact of disease for this domain [15]. Based on data from a large cohort, the Wien group proposed the Disease Activity in REActive Arthritis (DAREA) [16] composite measure, reintroducing successively it as Disease Activity for PSoriatic Arthritis (DAPSA) [17] which assesses 68 joints for tenderness and 66 joints for swelling. More recently, Psoriatic Arthritis Disease Activity Score (PASDAS) was developed [18]. Compared with existing indices, PASDAS has demonstrated having a better discriminate capacity in distinguishing high and low disease activity.

The aim of the present study was to apply and compare the performance of various composite activity indices in a cohort of patients with established peripheral PsA. The findings may serve as a reference to define levels of activity and response to be expected in the “real life” setting without any formal or financial restrictions.

## 2. Material and Methods

### 2.1. Patients

One hundred seventy-one patients with diagnosis of PsA, according CASPAR (classification criteria for psoriatic arthritis) [19], were enrolled in the study. Peripheral arthritis was considered present, if there had ever been tender and swollen joints assessed by a rheumatologist. Polyarthritis was defined as five or more inflamed (swollen or tender) joints as suggested by Helliwell et al. [20]. Patients with axial disease (presence of typical inflammatory back pain in combination with clinical signs of enthesitis or sacroiliitis) were excluded by our study. Further exclusion criteria were active skin disease other than psoriasis that would interfere with the assessment of a target lesion, other active concomitant musculoskeletal diseases, history of cancer or lymphoproliferative disease, uncontrolled diabetes, unstable ischemic heart disease, congestive heart failure, active inflammatory bowel disease, positive serology for hepatitis B, and history of active tuberculosis. Additionally, we excluded patients who fulfilled the classification criteria for fibromyalgia because the composite indices could be flawed. Considering that it was not a randomised trial, drug therapy was chosen by the managing clinician as it was considered appropriate [21]. All patients were attending the outpatient and inpatient clinics of the Rheumatology Department of the Università Politecnica delle Marche (Ancona, Italy) and they represent a “real life” sample of PsA referred to our department. The study was approved by the Hospital Clinic Ethics Committee. All patients agreed to be enrolled and provided signed informed consent.

### 2.2. Study Variables

A comprehensive questionnaire package, including sociodemographic data, quality of life items, and disease-related variables, was administered to the patients. The sociodemographic variables included age and gender. Disease-related characteristics included disease duration (years since fulfilment of the classification criteria of the PsA), comorbidity, and composite score used to evaluate the disease activity. We have chosen domains and instruments that have, in general, performed well in previous studies and were chosen by GRAPPA members and established at the various OMERACT conferences [22] as being essential components of psoriatic disease documentation. These evaluations of PsA include the following domains: peripheral joint assessment (68 joints for tenderness (68 TJC); 66 joints for swelling (66 SJC)), patient reported of pain on an 11-point numerical rating scale (NRS), physician and patient assessments of disease activity (PhGA and PtGA, resp.) on an 11-point NRS, patient general health status (GH on an 0–100 NRS), and dactylitis; (a simple dactylitic digit count was applied), Leeds Enthesitis Index (LEI) [23], physical function (e.g., as measured by the Health Assessment Questionnaire (HAQ) [12] and by physical component summary score (PCS) of the Medical Outcome Survey Short Form-36 (SF-36)) [24], a measure of severity of psoriatic lesions (as evaluated by the Psoriasis Area and Severity Index (PASI)) and of the impact of skin disease (e.g., as measured by the Dermatology Life Quality Index (DLQI)) [12, 25], acute phase reactants that is C-reactive protein (CRP) and erythrocyte sedimentation rate (ESR) and rheumatoid factor (RF, by nephelometry). The 68 tender and 66 swollen joints counts include: the temporomandibular, sternoclavicular, acromioclavicular, shoulder, elbow, wrist, metacarpophalangeal (MCP), PIP, DIP, hip, knee, talotibial, midtarsal (including subtalar), metatarsophalangeal, and interphalangeal joints of the toes (proximal and distal joints of each toe counted as one unit). Consensus concerning joint assessment was met to avoid high interrater variabilities among the physicians. The LEI is the only measure developed specifically for PsA [23] and includes an assessment of 6 sites: bilateral Achilles tendon insertions, medial femoral condyles, and lateral epicondyles of the humerus. Tenderness at each site was quantified on a dichotomous criteria (0 = not tender; 1 = tender). The HAQ, originally developed to assess disability in RA, by focusing on physical disability has been used widely in inflammatory arthritis clinical trials, including PsA. The DLQI was developed to measure the disability experienced by patients with different dermatologic conditions. Further, information about physical function was obtained with a validated Italian translation of the SF-36 [24]. The DLQI consists of 10 questions. Each question is scored individually on a scale of 0–3. Individual scores are summed to produce a total score ranging from 0 to 30. Higher scores reflect a greater influence on quality of life [25]. The DLQI has been validated in assessment of psoriasis and have shown discrimination and responsiveness in PsA trials [25]. These variables were used to calculate fulfilment of the MDA and remission criteria and all composite disease activity indices. A patient was classified as achieving MDA when meeting 5 of the 7 following criteria: 68 TJC ≤ 1; 66 SJC ≤ 1; PASI ≤ 1; NRS-pain ≤ 1.5; PtGA of disease activity ≤ 2; HAQ ≤ 0.5; tender entheseal points ≤ 1 [26]. The remission was defined according to previously published studies as absence of any swollen or tender joints as well as ESR < 20 mm during first hour and CRP < 0.5 mg/dL [27].

### 2.3. Composite Indices

In order to measure PsA disease activity, the main available composite scores such as DAS28 based on ESR (DAS28-ESR) [28] or on CRP (DAS8-CRP) [29], SDAI [11], CPDAI [15], DAPSA [17, 30], and PASDAS [18] have been calculated.

The DAS28 includes 28-SJC and 28-TJC in addition to GH scale and ESR values [29] and can be calculated by entering these four variables into the WEB calculator obtained from http://www.das-score.nl/das28/DAScalculators/dascalculatros.html. Further, a DAS28 based on CRP levels rather than ESR has been suggested [29]. The DAS28 (CRP) has been validated against radiographic progression and physical function. The DAS-CRP combines information from the 28-SJC and 28-TJC in addition to GH scale and CRP (in mg/L). DAS28-ESR can range from 0.49 to 9.07, whereas DAS28-CRP can range from 0.96 to 8.79.

The SDAI employs a linear sum of five untransformed unweighted variables, including 28-SJC and 28-TJC, PtGA and EGA, and CRP (in mg/dL). The range of SDAI is 0–86 [11].

The CPDAI is a domain-based measure which includes an evaluation of peripheral arthritis (66 SJC and 68 TJC), functional disability (HAQ), skin (PASI and DLQI), dactylitis (a simple count of each digit involved), enthesitis, and spinal manifestations [15]. For each domain, instruments are used to assess both the extent of disease activity and the effect of involvement in that domain on patients' function and health-related quality of life. For the purpose of our research, since we have been concerned only with patients with peripheral involvement, we excluded from our assessment the domain related to axial diseases. The modified CPDAI (mCPDAI) domains were scored using a 4-point scale from 0 (no disease activity) to 3 (most severe disease activity), giving an mCPDAI score range of 0–12 [30].

The DAPSA was adapted and renamed from the DAREA, a score validated for reactive arthritis [16]. It was developed from a clinical cohort and validated using clinical trial data [17]. It is composed of five untransformed, unweighted variables, including two patient-centered items (PtGA and pain on an 11-point NRS), one physician centered item (66-SJC), one item dependent on patient and physician (68-TJC), and a laboratory variable (CRP in mg/dL). Four of these variables (TJC, SJC, PtGA, and pain) were also ranked as key outcomes in OMERACT surveys.

The PASDAS was developed by multiple linear regression analysis [18]. It includes seven domains: evaluator and patient assessments of disease activity (PhGA and PtGA, resp.) on an 11-point NRS, skin, peripheral joint counts, dactylitis, enthesitis, acute phase response, and SF-36-PCS. The PASDAS is computed by the following equation: PASDAS = (((0,18∗∗√(PhGA) + 0.159∗∗√(PtGA) − 0.253∗∗√(SF36 − PCS) + 0.101∗log⁡_nat_(SJC66 + 1)) + 0.048∗log⁡_nat_(SJC68 + 1)) + 0.23∗log⁡_nat_  (Leeds  enthesitis  index + 1)) + 0.37∗log⁡_nat_  (tender  dactylitis  count + 1) + 0.102∗log⁡_nat_  (CRP + 1) + 2∗1.5.

### 2.4. Statistical Analysis

Continuous data were presented as means with standard deviations (SDs) or medians with 95% confidence interval (95% CI), depending on the distribution of the data (tested with the Kolmogorov-Smirnov test). Histograms were used to visualise the distribution of the scores. Categorical data were presented as proportions. Demographic and clinical measures were compared using Mann-Whitney *U* test or Kruskal-Wallis test for continuous variables and chi-square analysis for discontinuous variables. To evaluate discriminative performance in terms of the ability of the indices to discriminate between patients in different activity grades, the receiver operating characteristic (ROC) curve analysis was used. The criteria for MDA and for remission were applied as external criterion. The area under the ROC curve (AUC) was calculated to quantify the discriminative performance. From the ROC curves, the optimal cut-off point corresponding to the maximum sum of sensitivity and specificity was computed. The nonparametric Wilcoxon's signed ranks test is used for calculation and comparison of the areas under the ROC curves. The convergent validity by correlating the scores of the composite indices with the other measures applied in the study was examined. A particular variable is expected to converge with the scores of those instruments targeting the same construct and to deviate from the scores given by instruments or scales assessing a different one (divergent validity). To quantify these relationships, Spearman's rho correlation coefficients were obtained. *P* values below 0.05 were regarded as statistically significant. All data were entered into a Microsoft Access database, which had been developed for management of cross-sectional study. The data were analysed using the SPSS version 11.0 (SPSS Inc, Chicago, IL), and the MedCalc version 16.0 (MedCalc Software, Mariakerke, Belgium).

## 3. Results

### 3.1. Patient Characteristics

One hundred seventy-one patients (98 women, 73 men) fulfilling the CASPAR criteria [19] are included in the study. The PsA cohort included oligoarticular disease (56.1%) and polyarticular disease (43.9%). Age at inclusion was slightly, but not significantly, lower in men (50.4 ± 13.1 versus 52.8 ± 15.3 years, *P* = 0.07). Disease duration before inclusion was similar in men and women (7.1 versus 7.8 years). RF was positive in 1.7% of women and 2.8% of men (not significant).  Table 1 provides detailed demographic, clinical, and laboratory data of all patients. These data are expressed as mean standard deviation and medians (95% CI). As expected from previous cohort studies [9], the PsA patients tended to be younger males and had lower joint counts and disease activity scores in comparison to RA patients. More than 90% of patients had at least one comorbid condition mostly of a metabolic or cardiovascular nature. Most subjects had more than one comorbid condition with a median number of 3 (range from 1 to 4). One hundred fifty-one patients (88.3%) with PsA were taking disease-modifying antirheumatic drugs (DMARDs) and/or biologic agents such as methotrexate, leflunomide, sulfasalazine, adalimumab, and infliximab; 67 patients (39.2%) with PsA were additionally treated with low-dose corticosteroids (<10 mg/day of prednisolone). Additional drug therapy included NSAIDSs on an on-demand basis and analgesics, such as acetaminophen. Moreover, local skin treatment comprising corticosteroid preparations was administered in 79 patients (46.2%).

### 3.2. Descriptive Statistics of Composite Disease Activity Indices

**Figure 1:**
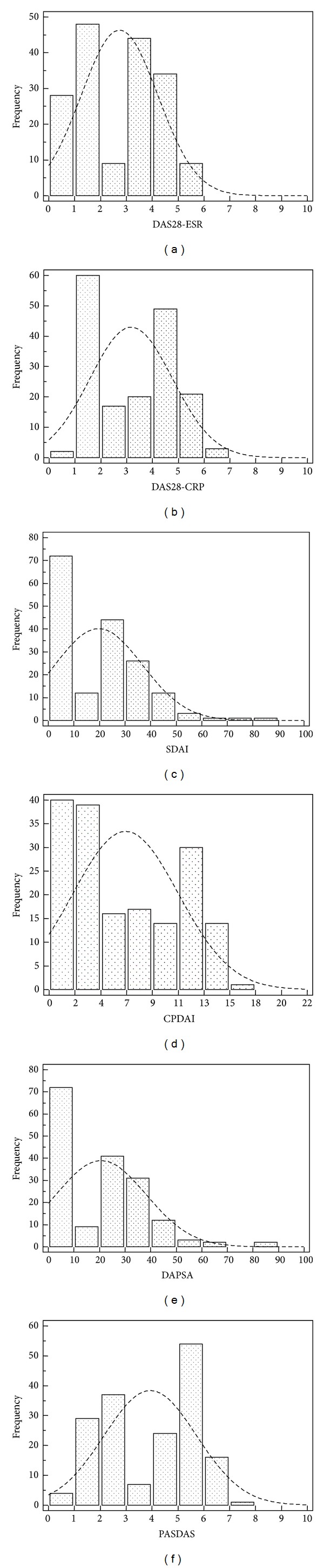
Estimates of central tendency and distributions of composite measures evaluated in the study. The bar on the left of each group represents the number of subjects with a score of 0 (floor effect). The bar on the right represents the number of subjects with a maximum possible score (ceiling effect).

Table 2 summarizes the descriptive statistics of all composite disease activity indices.  Figure 1 shows estimates of central tendency and distributions for all the composite measures included in the study. All composite scores were not normally distributed (Kolmogorov-Smirnov test) and the distribution in all cases was a bimodal type, probably related to the different type of cases enrolled (56.1% oligoarticular and 43.9% polyarticular). The medians (95% CI) were as follows: DAS28-CRP 3.59 (2.37 to 4.00), DAS28-ESR 3.13 (1.87 to 3.52), SDAI 21.00 (10.84 to 25.53), CPDAI 5.00 (4.00 to 7.00), DAPSA 22.05 (11.51 to 26.73), and PASDAS 4.21 (3.39 to 4.88) (Table 2).

### 3.3. Discriminant Validity

The ROC curves were similar for the two categories of composite measures. The discriminatory MDA power of RA specific composite indices such as DAS28 CRP, DAS28-ESR, and SDAI and PsA specific composite indices such as CPDA, DAPSA, and PASDAS was very good. They did not show significant differences: AUC of 0.894 (95% CI 0.838 to 0.936) for DAS28 CRP; 0.892 (95% CI 0.836 to 0.934) for DAS28-ESR; 0.902 (95% CI 0.847 to 0.942) for SDAI; 0.792 (95% CI 0.724 to 0.9850) for CPDAI; 0.899 (95% CI 0.843 to 0.939) for DAPSA; and 0.877 (95% CI 0.818 to 0.922) for PASDAS (Table 1 (supplementary file) shows the discriminatory power of all PsA composite indices for MDA and remission). Similar results were observed regarding their discriminatory power in terms of remission (Table 1, Supplementary Material available online at http://dx.doi.org/10.1155/2014/528105). Briefly: AUC of 0.886 for DAS28 CRP, 0.891 for DAS28-ESR, 0.905 for SDAI, 0.818 for CPDAI, 0.896 for DAPSA, and 0.882 for PASDAS. All differences between areas were not significant.

**Figure 2:**
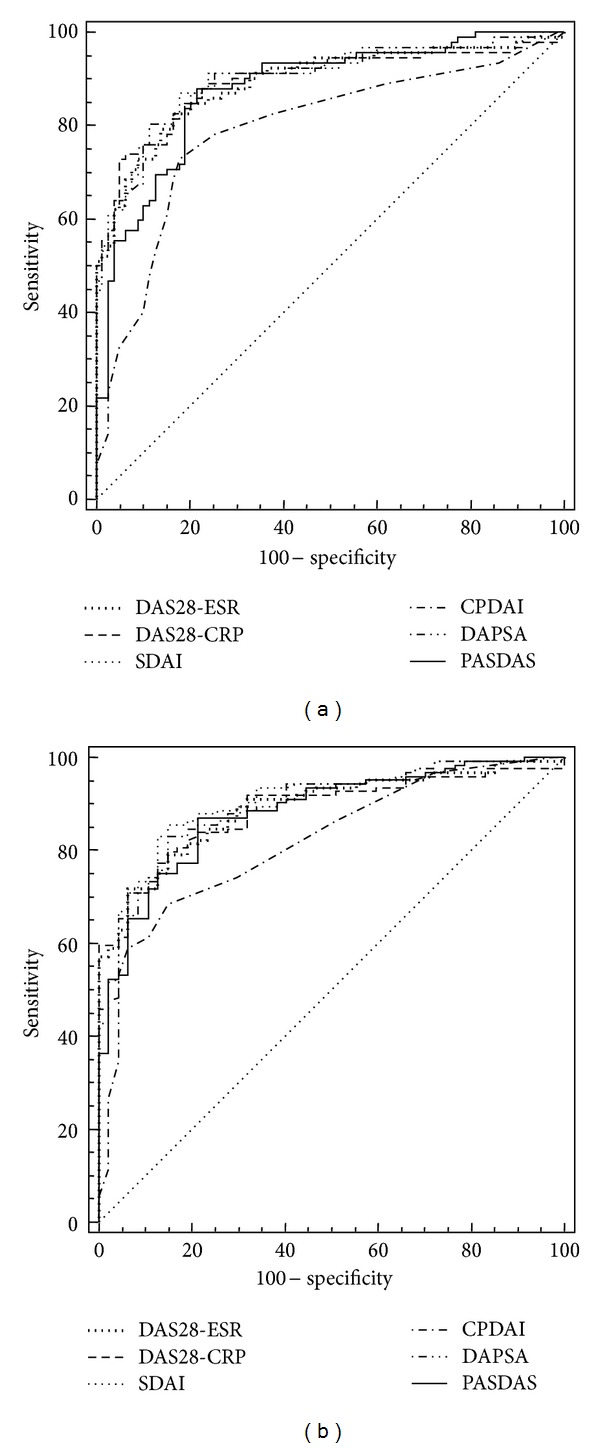
ROC curves for the discriminatory MDA and remission power of composite disease activity indices.

Figure 2 shows the ROC curves for the discriminatory MDA and remission power of composite disease activity indices in PsA patients. From these data, we obtained the list of sensitivity and specificity and the relative value of likelihood ratio (LR) for the possible threshold values, and we chose those with the highest diagnostic accuracy (minimal false negative and false positive results) (Table 3).

**Figure 3:**
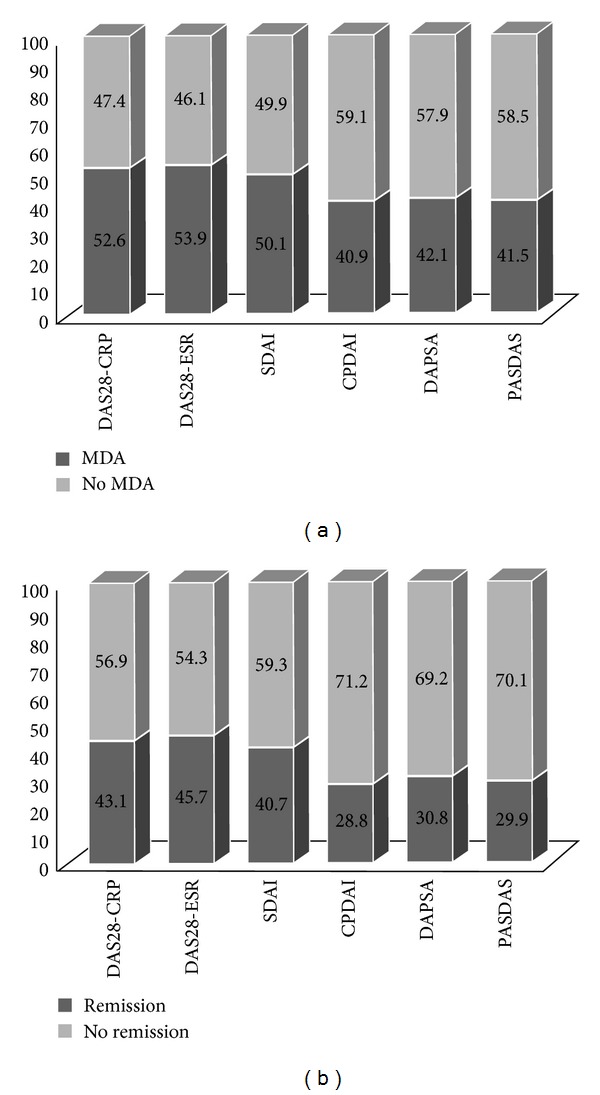
(a) Proportion of patients in or not in minimal disease activity (MDA) according to various composite indices. (b) Proportion of patients in or not in clinical remission according to various composite indices.

These data showed that the DAS28-ESR and DAS28-CPR cut-off points, required to evaluate achievement of MDA and remission, were below and more stringent (3.6 and 2.4, resp.) than those approved by the EULAR for RA [9]. The cut-off points for the SDAI approved both by ACR and EULAR for RA were similar to those for PsA (3.3 and 11, resp.) [14]. Moreover, with regard to CPDAI, DAPSA, and PASDAS, we have calculated cut-off values to define MDA (5, 15, and 3.6, resp.) and remission (2, 4, and 2.4, resp.) (Table 3). The sensitivity values were found for all indices to be slightly high (range 66.5 to 79.3), while those relating to the specificity were reasonably high (range 83.5 to 90.2). Positive likelihood ratios were similar in the definition of MDA (range 4.2 to 7.1) and remission (4.6 to 6.9). Using these cut-off values calculated by ROC analysis, CPDAI, DAPSA, and PASDAS had the most stringent definitions of MDA (40.9%, 42.1%, and 41.5%, resp.) and remission (28.8%, 30.8%, and 29.9%, resp.) (Figures 3(a)-3(b)). DAS28-ESR and DAS28-CRP had the highest proportions in MDA (52.6% and 53.9%, resp.) and remission (43.1% and 45.7%, resp.). SDAI showed an intermediate response (40.7% for remission and 50.1% for MDA).

### 3.4. Concurrent Validity

There was a very high degree of correlation between the composite indices. The indices were correlated significantly with all other composite comparator scores (all *P* levels < 0.0001) (Table 2, supplementary file). The highest correlations were seen between DAPSA and SDAI (rho = 0.996) and between DAPSA and DAS28-CRP (rho = 0.957). The CPDAI, DAPSA, and PASDAS were all significantly correlated with HAQ at levels of rho of 0.760, 0.828, and 0.842, respectively, with SF-36-PCS at levels of rho of −0.708, −0.693, and −0.792, respectively, and with DLQI at levels of rho = 0.567, 0.622, and 0.628, respectively (all at *P* levels < 0.0001). Significant high correlations (*P* < 0.0001) were also seen between CPDAI, DAPSA, and PASDAS and other self-reported measures, such as ratings of pain (rho = 0.761, 0.713, and 0.857, resp.), PhGA (rho = 0.781, 0.789, and 0.932, resp.), and PtGA (rho = 0.782, 0729, and 0.934, resp.). Significant, but less, robust correlations were found with CRP (rho = 0.481, 0.655, and 0.693, resp.) and ESR (rho = 0.431, 0.605, and 0.613, resp.). The CPDAI, DAPSA, and PASDAS showed no significant relationship with age, gender, and disease duration.

## 4. Discussion

To date, there is still no consensus about what clinical tool should be used to measure adequately the global features of PsA. The core domains and tools to be used both in clinical trials and care in PsA patients have been identified by GRAPPA and preliminary validation was obtained through the OMERACT process [13]. These domains can be assessed by single and composite measures. For assessing peripheral joint arthritis in PsA, some indices have been “borrowed” from RA and adapted to PsA, whilst other measures have been developed specifically for PsA.

It is clear that composite measures used in RA, such as the DAS28 or the SDAI, assess disease activity only in 28 joints and, therefore, do not fully represent all aspects of peripheral psoriatic disease. Moreover, these composite measures do not fully evaluate the multiple clinical domains of PsA (e.g., enthesitis, dactylitis, and skin involvement). Ideally, any composite measure should retain the ability to be broken down into its disparate domains, as is the case with the CPDAI, so that the effects of each of these individual aspects of the disease and their potential for differential treatment response can be assessed. Composite measures may also need sophisticated weighting of the various components such as in the case of PASDAS which is specific to psoriatic disease. Although the potential value of these measures in PsA had not been definitely clarified, Fransen and Van Riel [8] showed that EULAR response criteria performed better than the Psoriatic Arthritis Response Criteria (PsARC) in discriminating active from placebo drugs and that DAS and DAS28 performed better than single core-set measures in PsA. Furthermore, another study has reported that the DAS28 is a valid instrument for measuring disease activity with respect to response to biologic therapies [31]. Pooled indices are generally more responsive and performed better in discriminating active drug from placebo than the single core-set measures [15–18, 32].

Our study represents a first attempt to compare the discriminative capacity and constructed validity of existing composite measure of disease activity in patients with peripheral PsA. For this purpose, composite indices specifically developed and validated in RA (DAS28-ESR, DAS28-CPR, and SDAI) and in PsA (CPDAI, DAPSA, and PASDAS) were included. The ROC curves to test the ability of the different indices to discriminate between patients in different phases of activity were similar to the two categories of composite measures (those developed for RA and those proposed for PsA). Only the CPDAI showed less discriminative (but not statistically significant) ability than the other pooled indices. The analysis of convergent validity showed a significant correlation (*P* < 0.0001) between the six disease activity indices in terms of absolute scores. In addition, CPDAI, DAPSA, and PASDAS results are highly correlated with the individual clinical variables and functional measures of disease activity. Although a good concurrent validity and discriminant capacity of six disease activity indices were observed; the indices examined showed a difference among the numbers of patients categorized by the response criteria. In particular, the rate of patients in remission was clearly different among the respective criteria. CPDAI, DAPSA, and PASDAS showed the most stringent definitions of remission and MDA category. Both DAS28-ESR and DAS28-CRP are demonstrated to be less conservative in the evaluation of residual disease, whilst SDAI remission was slightly more conservative than DAS28 remission criteria.

Similarly, to the high rate of remission observed in our series using the DAS28 ESR and CRP, Saber et al. [33] have shown that the rate of remission of 12 months, defined according to the Disease Activity Score using DAS28-CRP, was achieved in 58% of PsA patients. While the 28-joint counts in RA assessment are well accepted and validated (despite the exclusion of the evaluation of the feet); in PsA, a more complete joint evaluation is needed. The DAS28 has been criticized in RA for its omission of the ankles and feet [34], and defining remission on the basis of the DAS28 (a DAS28 score < 2.6) has engendered controversy with regard to the bounding values [35], for which a more recent criterion has been proposed [36]. The acute phase reactants (CRP level and ESR) weigh heavily in the DAS28 calculation, which may erroneously lower the DAS28 score in the face of objective evidence of ongoing disease activity in the joints [37], especially since a significant proportion of patients with RA can have a normal ESR and normal level of CRP at presentation [38] including some patients with radiographic evidence of progressive erosive disease [39]. In this regard, the new 2011 ACR/EULAR RA criteria [40] recognize that residual disease activity can be present in the feet of patients deemed to be in remission, and the joint committee recommends but does not require the inclusion of the ankles and forefeet in assessment of remission [40, 41]. Moreover, in individual patients, excluding the feet and DIP joints from joint counts may lead to underestimation of disease activity. This is especially true in patients with monoarthritis or oligoarthritis and patients with predominantly, or exclusively, DIP involvement [33, 42, 43]. Although the changes observed in the DAS28 in a placebo-controlled trial in patients with PsA suggest that this clinical measure may be applicable in PsA [44], DAS28 or SDAI for patients with PsA appear more appropriate in RA assessment but not for oligoarticular disease. In addition, even though the squared-root transformation of the swollen and tender joint count minimizes in DAS28, the weight of the joint global value of the composite index, DAS28, and SDAI does not take into account typical features of PsA [27].

The RA scoring system did not appear appropriate to define “remission” in PsA; in terms of quantitative threshold, it does not capture the additional clinical features of PsA, enthesitis, dactylitis, and skin disease. GRAPPA and other groups have actively worked to validate the composite disease activity score, addressing all clinical domains of PsA, and the fact that they were able to meaningfully capture the impact of each domain on outcomes [15].

A recent analysis using the Psoriasis Randomised Etanercept Study in Subjects with Psoriatic Arthritis (PRESTA) dataset compared the performance of the CPDAI and the DAPSA [45]. Analyses revealed that both the CPDAI and the DAPSA showed good responsiveness to change. CPDAI but not DAPSA identified a significant difference between treatment groups that were likely driven by the differential response in skin disease. In stepwise regression analysis, enthesitis, HAQ, dactylitis, and DLQI all contributed significantly to CPDAI values at baseline. Thus, while both the DAPSA and CPDAI show responsiveness in measures of arthritis, the CPDAI has a potential advantage in that it can also reflect changes in the other domains of PsA. More recently, Helliwell et al. [18], therefore, proposed the PASDAS as a composite of three visual analogue scales. The GRACE dataset further permitted a comparison of this proposed measure with the CPDAI, DAPSA, and DAS28; all 4 measures demonstrated adequate discrimination in terms of the construct of disease activity. In addition, all these measures showed good responsiveness in the GRACE dataset.

Limitations to our study are seen in addition to specific limitations of each analytic method. In particular, the main limit of the study is related to the “circularity” of the method. Considering that MDA emphasize one dimension of disease in PsA, the articular component, this measure will match more closely purely articular indices such as DAS28, DAPSA, or CPDAI and could make the interpretation of the optimal cut-off levels problematic [46, 47]. This could lead to the identification of different cut-off levels than those that we identified, helping to keep the discussion about this topic open. The different methodology used by GRAPPA for the development of ASDAS may add clarifying elements with this regard [46].

A second limitation is the cross-sectional design which does not allow the evaluation of the sensitivity to change of the indices. Further, this study was performed in a single centre within a relatively small region. Finally, in the study population, despite being representative of the entire patient population with PsA, we excluded patients suffering from axial disease. However, in daily rheumatology care, highly active patients, as, for example, those included in clinical trials, are rarely seen. In spite of DAPSA being originally not directly derived from patients with PsA, the index has more performance in terms of discriminant capacity and constructed validity for the evaluation of disease activity in peripheral PsA. Moreover, it has higher face validity, since it employs a large joint count (66 swollen joint and 68 tender joint) rather than the 28-joint count used in the DAS28 an SDAI. Also, the simplicity of calculating DAPSA might be regarded as a further advantage, which makes it easy to use both in clinical trials and clinical practice. Further, a simple summation of disease activity variables without a sophisticated weighting of the various components certainly improves the feasibility in the period of time necessary to compute the index in routine settings but would not be able to discriminate between the magnitudes of activity of different domains. For this reason, the PASDAS, that contains all core domains identified for use in PsA clinical trials and provides a comprehensive assessment of disease activity, has been developed [18]. The main limitation could be related to the fact that PASDAS is time consuming, since it requires complex mathematical calculations to obtain a single score. However, this problem is still surmountable by using web- and/or calculator-based algorithms.

In conclusion, although a good concurrent validity and discriminant capacity among the six disease activity indices were observed, there is still contrasting data in the classification of patients according to the disease activity levels, and no decision on the optimal activity measures could be made without further work. A prospective validation study is currently underway to evaluate the metrologic properties of multimodal indices including ultrasound findings [48] as a more objective measure of disease activity in PsA.

## Bullet Points

- CPDAI, DAPSA, and PASDAS showed most stringent definitions of remission and MDA category, whereas DAS28-ESR and DAS28-CRP demonstrated to be less conservative in the evaluation of residual disease.
- Although a good concurrent validity and discriminant capacity of six disease activity indices were observed, these showed a difference among the numbers of patients categorized by the response criteria.
- In spite of DAPSA being originally not directly derived from patients with PsA, the index has more performance in terms of discriminant capacity and constructed validity for the evaluation of disease activity in peripheral PsA.

## Supplementary Material

Table I: Discriminatory power of composite indices for MDA and remission.
Table II: Correlation between the different composite indices evaluated in the study.

## Figures and Tables

**Table 1:** Demographic, clinical, and laboratory data of study population.

	Mean	SD	Median	25–75 **P**
Demographic data				
Age (yrs)	51.55	11.61	51.00	42.50–59.00
Gender (M/F)				
Duration psoriatic arthritis (yrs)	7.48	4.96	6.00	3.00–11.00
Duration of psoriasis (yrs)	13.87	6.98	12.00	7.00–16.00
Acute phase reactants				
ESR (mm/h)	27.50	28.48	22.00	13.00–34.00
CRP (m/dL)	4.42	7.06	1.55	0.54–4.88
Enthesis/dactylitis counts				
Leeds Enthesitis Index (range 0–6)	1.02	1.24	1.00	0.00–1.50
Dactylitis count (range 0–20)	1.04	1.07	1.00	0.00–2.00
Skin				
Psoriasis area and severity index (score 0–72)	5.60	4.93	4.30	1.80–8.40
Peripheral joint counts				
28 tender joint count	4.46	4.45	4.00	0.00–8.00
28 swollen joint count	2.02	2.24	2.00	0.00–4.00
68 tender joint count	6.06	5.97	4.50	0.50–11.00
66 swollen joint count	2.80	2.99	2.00	0.00–5.00
Patient/physician NRS scores				
Pain (0–10)	35.46	24.96	40.00	10.00–55.00
Fatigue (0–10)	32.82	24.37	35.00	10.00–55.00
PtGA (0–10)	4.10	3.04	4.0	1.0–6.5
EGA (0–10)	3.56	2.55	4.0	1.0–6.0
GH (0–100)	36.58	21.74	4.0	0–100
Health-related quality of life and function				
SF36 PCS	40.47	9.08	39.87	33.43–47.92
SF36 MCS	42.00	11.40	40.51	32.87–51.04
DLQI (0–30)	9.22	7.02	7.00	3.00–16.00
HAQ (0–3)	0.92	0.62	0.87	0.36–1.50
Composite activity indices				
DAS28-CRP	3.17	1.59	3.59	1.44–4.46
DAS28-ESR	2.73	1.48	3.13	1.11–3.99
SDAI	19.69	17.07	21.00	3.14–30.94
CPDAI	6.52	4.49	5.00	3.00–11.00
DAPSA	20.62	17.64	22.05	3.45–32.40
PASDAS	3.93	1.78	4.21	2.18–5.49

ESR: erythrocyte sedimentation rate; CRP: C-reactive protein; PtGA: disease activity patient's assessment; EGA: disease activity evaluator's assessment; GH: global health status; SF36 PCS: physical component summary score of the Medical Outcome Survey Short Form-36; SF36MCS: mental component summary score of the Medical Outcome Survey Short Form-36; DLQI: Dermatology Life Quality Index; HAQ: Health Assessment Questionnaire; DAS28: 28-Disease Activity Score; SDAI: Simplified Disease Activity Index; CPDAI: Composite Psoriatic Disease Activity Index; DAPSA: disease activity in psoriatic arthritis; PASDAS: Psoriatic Arthritis Disease Activity Score.

**Table 2:** Descriptive statistics of all composite disease activity indices.

	DAS28-CRP	DAS28-ESR	SDAI	CPDAI	DAPSA	PASDAS
Lowest value	**0.99**	**0.70**	**0.40**	**0.00**	**0.40**	**0.34**
Highest value	**6.22**	**5.54**	**88.10**	**12.00**	**89.10**	**7.18**
Arithmetic mean	3.17	2.73	19.69	6.16	20.62	3.93
95% CI for the mean	2.93 to 3.41	2.51 to 2.95	17.12 to 22.26	5.57 to 6.76	17.96 to 23.28	3.66 to 4.20
Median	3.59	3.13	21.00	5.00	22.05	4.21
95% CI for the median	2.37 to 4.00	1.87 to 3.52	10.84 to 25.53	4.00 to 7.00	11.51 to 26.73	3.39 to 4.88
Variance	2.55	2.19	291.69	15.81	311.37	3.19
Standard deviation	1.59	1.48	17.07	3.97	17.64	1.78
Relative standard deviation	0.50 (50.32%)	0.54 (54.18%)	0.86 (86.72%)	0.64 (64.47%)	0.85 (85.55%)	0.45 (45.46%)
Standard error of the mean	0.12	0.11	1.30	0.30	1.34	0.13
Coefficient of skewness	0.04 (*P* = 0.8220)	0.04 (*P* = 0.8131)	0.84 (*P* < 0.0001)	0.26 (*P* = 0.1502)	0.79 (*P* = 0.0001)	−0.16 (*P* = 0.3548)
Coefficient of kurtosis	−1.46 (*P* < 0.0001)	−1.54 (*P* < 0.0001)	0.86 (*P* = 0.0509)	−1.44 (*P* < 0.0001)	0.71 (*P* = 0.0887)	−1.42 (*P* < 0.0001)
Kolmogorov-Smirnov test for normal distribution	reject Normality (*P* = 0.0020)	reject Normality (*P* = 0.0007)	reject Normality (*P* < 0.0001)	reject Normality (*P* < 0.0001)	reject Normality (*P* = 0.0001)	reject Normality (*P* = 0.0014)

ESR: erythrocyte sedimentation rate; CRP: C-reactive protein; DAS28: 28-Disease Activity Score; SDAI: Simplified Disease Activity Index; CPDAI: Composite Psoriatic Disease Activity Index; DAPSA: disease activity in psoriatic arthritis; PASDAS: Psoriatic Arthritis Disease Activity Score.

**Table 3:** Criterion values and coordinates of the ROC curve for MDA and remission criteria according to the different composite indices.

Composite activity indices	Optimal cut-off value	Sensitivity	95% CI	Specificity	95% CI	+LR	95% CI	−LR	95% CI
Minimal disease activity									
DAS28-CRP	≤3.6	76.09	66.1–84.4	88.75	79.7–94.7	6.76	3.6–12.6	0.27	0.2–0.4
DAS28-ESR	≤3.6	72.83	62.6–81.6	88.75	79.7–94.7	6.47	3.5–12.1	0.31	0.2–0.4
SDAI	≤11	79.35	69.6–87.1	88.75	79.7–94.7	7.05	3.8–13.2	0.23	0.2-0.3
CPDAI	≤5	68.48	58.0–77.8	83.54	73.5–90.9	4.16	2.5–7.0	0.38	0.3–0.5
DAPSA	≤15	77.17	67.2–85.3	88.75	79.7–94.7	6.86	3.7–12.8	0.26	0.2–0.4
PASDAS	≤3.6	76.09	66.1–84.4	86.25	76.7–92.9	5.53	3.2–9.7	0.28	0.2–0.4
Remission									
DAS28-CRP	<2.4	72.00	63.3–79.7	89.36	76.9–96.5	6.77	2.9–15.6	0.31	0.2–0.4
DAS28-ESR	<2.4	71.20	62.4–78.9	88.86	76.1–96.1	6.69	2.8–15	0.32	0.2–0.4
SDAI	<3.3	73.60	65.0–81.1	90.16	77.1–96.9	6.92	3.0–15.9	0.30	0.2–0.4
CPDAI	<2	68.55	59.6–76.6	85.11	71.7–93.8	4.60	2.3–9.2	0.37	0.3–0.5
DAPSA	<4	72.80	64.1–80.4	89.36	76.9–96.5	6.84	3.0–15.8	0.30	0.2–0.4
PASDAS	<2.4	75.20	66.7–82.5	85.11	71.7–93.8	5.05	2.5–10.1	0.29	0.2–0.4

LR: likelihood ratio; 95% CI: 95% confidence intervals; ESR: erythrocyte sedimentation rate; CRP: C-reactive protein; SF36 PCS: physical component summary score of the Medical Outcome Survey Short Form-36; SF36MCS: mental component summary score of the Medical Outcome Survey Short Form-36; DLQI: Dermatology Life Quality Index; HAQ: Health Assessment Questionnaire; DAS28: 28-Disease Activity Score; SDAI: Simplified Disease Activity Index; CPDAI: Composite Psoriatic Disease Activity Index; DAPSA: disease activity in psoriatic arthritis; PASDAS: Psoriatic Arthritis Disease Activity Score.
