# Supplementary material for: Disease Activity in Psoriatic Arthritis: Comparison of the Discriminative Capacity and Construct Validity of Six Composite Indices in a Real World
Source: Biomed Res Int. 2014 May 20;2014:528105. doi: 10.1155/2014/528105 (PMC4055291; doi:10.1155/2014/528105)
Supplement: Supplementary file 1 — Table I: Discriminatory power of composite indices for MDA and remission. Table II: Correlation between the different composite indices evaluated in the study. [file 528105.f1.doc]

**Table I (supplementary file)**. Discriminatory power of composite indices for MDA and remission.

| **Composite activity indices** | **AUC** | | **SE ^a^** | | **95% CI ^b^** |
| --- | --- | --- | --- | --- | --- |
| **Minimal Disease Activity** | | | | | |
| DAS28-CRP | 0.886 | 0.0251 | | 0.828 to 0.929 | |
| DAS28-ESR | 0.891 | 0.0245 | | 0.834 to 0.933 | |
| SDAI | 0.905 | 0.0237 | | 0.851 to 0.945 | |
| CPDAI | 0.818 | 0.0343 | | 0.752 to 0.872 | |
| DAPSA | 0.896 | 0.0247 | | 0.840 to 0.937 | |
| PASDAS | 0.882 | 0.0268 | | 0.824 to 0.926 | |
| **Remission** | | | | | |
| DAS28-CRP | 0.894 | 0.0255 | | 0.838 to 0.936 | |
| DAS28-ESR | 0.892 | 0.0250 | | 0.836 to 0.934 | |
| SDAI | 0.902 | 0.0237 | | 0.847 to 0.942 | |
| CPDAI | 0.792 | 0.0355 | | 0.724 to 0.850 | |
| DAPSA | 0.899 | 0.0240 | | 0.843 to 0.939 | |
| PASDAS | 0.877 | 0.0266 | | 0.818 to 0.922 | |

*^a^ Hanley & McNeil, 1982; ^b^ Binomial exact*

*ESR = erythrocyte sedimentation rate; CRP = C-reactive protein; DAS28 = 28-Disease Activity Score; SDAI = Simplified Disease Activity Index; CPDAI = Composite Psoriatic Disease Activity Index; DAPSA = Disease Activity in Psoriatic Arthritis ; PASDAS = Psoriatic Arthritis Disease Activity Score.*

**Table II (supplementary file).** Correlation between the different composite indices evaluated in the study.

|  | | **DAS28-CRP** | | **DAS28-ESR** | | **SDAI** | | **CPDAI** | | **DAPSA** | | **PASDAS** | |
| --- | --- | --- | --- | --- | --- | --- | --- | --- | --- | --- | --- | --- | --- |
| **DAS28-CRP** | Correlation Coefficient Significance Level P | | ------  ------ | | 0.946 <0.0001 | | 0.947 <0.0001 | | 0.730 <0.0001 | | 0.957 <0.0001 | | 0.910 <0.0001 |
| **DAS28-ESR** | Correlation Coefficient Significance Level P | |  | |  | | 0.933 <0.0001 | | 0.740 <0.0001 | | 0.933 <0.0001 | | 0.905 <0.0001 |
| **SDAI** | Correlation Coefficient Significance Level P | |  | |  | |  | | 0.676 <0.0001 | | 0.996 <0.0001 | | 0.884 <0.0001 |
| **CPDAI** | Correlation Coefficient Significance Level P | |  | |  | |  | |  | | 0.686 <0.0001 | | 0.831 <0.0001 |
| **DAPSA** | Correlation Coefficient Significance Level P | |  | |  | |  | |  | |  | | 0.887 <0.0001 |
| **PASDAS** | Correlation Coefficient Significance Level P | |  | |  | |  | |  | |  | | ------  ------ |

*ESR = erythrocyte sedimentation rate; CRP = C-reactive protein; DAS28 = 28-Disease Activity Score; SDAI = Simplified Disease Activity Index; CPDAI = Composite Psoriatic Disease Activity Index; DAPSA = Disease Activity in Psoriatic Arthritis ; PASDAS = Psoriatic Arthritis Disease Activity Score.*
